# The impact of Muslim and Christian religious leaders responding to COVID-19 in Israel

**DOI:** 10.3389/fpubh.2022.1061072

**Published:** 2022-12-13

**Authors:** Jumanah Essa-Hadad, Nour Abed Elhadi Shahbari, Daniel Roth, Anat Gesser-Edelsburg

**Affiliations:** ^1^Department of Population Health, Azrieli Faculty of Medicine, Bar-Ilan University, Safed, Israel; ^2^Health and Risk Communication Lab, School of Public Health, University of Haifa, Haifa, Israel; ^3^Mosaica - Religion, Society, and State, Jerusalem, Israel; ^4^Program for Conflict Management and Negotiation, Bar-Ilan University, Ramat Gan, Israel

**Keywords:** COVID-19, religious leaders, health promotion, community engagement, health policy makers

## Abstract

**Background:**

The COVID-19 pandemic is one of the most significant public health emergencies in decades and has affected all countries worldwide. Religious leaders have been recognized as playing a pivotal role in health promotion during times of crisis. This study explored the role that Muslim and Christian religious leaders played in Israel during the pandemic, and the impact that their activities had on the community.

**Methods:**

A qualitative study involving semi-structured interviews was conducted with Muslim and Christian religious leaders and health policy makers from the Arab community. Intensive purposeful sampling was used to locate the two target audiences. Interview protocols were developed and included questions about the role they played during the pandemic, challenges they faced, and dialogue and partnerships they had. Interviews were audio-recorded, transcribed, and analyzed using thematic content analysis.

**Results:**

Ten Muslim Sheikhs, three Christian clergy, and four health policy makers were interviewed. Religious leaders played a predominant role in promoting health during the COVID-19 crisis. Both religious leaders and health policy makers reported similar challenges including dealing with fake news and the conspiracy theory, social events and gatherings, frustrations about gaps in policy toward religious institutions, and lack of trust toward State. Health policy makers recognized the key role religious leaders played and emphasized the importance of engaging religious leaders.

**Conclusion:**

The COVID-19 pandemic has been a catalyst for partnership between health policy makers and religious leaders. Religious leaders should play an integral and integrated role in promoting health during future health crises, not only in implementation of guidelines but also in development of policy so that the guidelines are tailored and sensitive to specific communities to avoid conflicts. As trusted authorities, religious leaders serve as a bridge between health authorities and communities and can be mediators who reconcile science, policy and religious perspectives. The routine cooperation between decision makers, opinion leaders, and religious leaders as social gatekeepers can increase the public's level of trust in the system.

## Introduction

On March 11, 2020, the WHO declared a global pandemic due to the spread of the COVID-19 virus (1). The COVID-19 pandemic is one of the most significant public health crisis in decades and has affected in some way everyone worldwide. It has had an adverse impact on societies worldwide, with dire consequences not only on health, but on all walks of life, including economy, society, politics, and culture.

World history reports both positive and negative impacts on infectious diseases by religious norms, practices, and observances (2). Individuals have always been able to use religion to calm themselves in times of natural and unnatural disasters and calamities, and religion has always played a crucial role in easing their stress. The COVID-19 crisis strengthened religious faith and spirituality (3). Koenig (4) emphasized the role of religious practice and faith in maintaining good health during the COVID-19 pandemic (4). Even among secular cultures, spirituality was found to have an active and positive role in the quality of life and health during the pandemic (5). One systematic review conducted by de Diego-Cordero et al. (6) revealed the important role spirituality had during COVID-19 as a coping tool to reduce individuals' stress, anxiety, and depression while increasing resilience and feelings of hope (6). However, as reported in a scoping review implemented in the UK, due to restrictions and lockdowns, spiritual support especially for dying patients and their families was significantly reduced (7).

Historically, different religions have a different take on infectious diseases. However, in general, all religions are in favor of helping to improve conditions of the sick (8), even though there is a fine balance between pandemic control measures and religious behaviors and observances (9). Particularly in countries with traditional societies, religious leaders have been acknowledged as key stakeholders in community engagement activities (2). Religious leaders can play an integral role during times of crisis, particularly in addressing challenges around religious gatherings and events, building trust of the community, promoting effect communication and advocacy, identifying and responding to the unique needs of the community, and promoting specific behaviors (10, 11). Religious leaders significantly help provide health information and are often more trusted than political leadership (12, 13). In the past, during times of health crises, religious leaders have served as community gatekeepers, since they have direct access to the community and are well-respected and trusted by both their followers, as well as health care professionals and State institutions. The WHO, Centers for Disease Control and Prevention (CDC), many national governments, and other actors have learned from previous crisis emergencies, including the Ebola epidemic, of the potential positive impact and engagement that religious leaders can have when properly mobilized (14). On the other side, some religious leaders view faith as opposed to science, which can actually exacerbate the situation. It is critical for public health authorities to engage religious leaders, working together on effective responses to pandemics (15).

Some studies have been conducted to investigate the relationship and involvement of religious leaders and clergy during the COVID-19 pandemic worldwide. One study carried out in New Zealand found that spiritual leaders and communities experienced four challenges during the restriction and lockdown period, mainly worship practices and maintaining physical distancing during religious practices (15). In Poland, religious congregations implemented a wide range of activities in the fight against the COVID-19 pandemic, including providing medical assistance and nursing within the health care system, charitable work (material, financial, and welfare/housing aid), and pastoral, religious, educational, psychological, and missionary activities (16). Research from Indonesia also showed that Islamic organizations helped mitigate the disease by providing medical, theological, and educational services (17). Another study conducted in Africa showed that faith-based organizations played a pivotal role in adapting and responding to health emergencies by providing care, particularly in rural settings (18). Religious leaders in a tight-knit Modern Orthodox Jewish community in New York played a vital role in promoting the wellbeing of their constituents by organizing community outreach, mobilizing tangible services, conducting virtual religious services, and relaying health-related information (19). In Christian communities in the Los Angeles area, the majority of churches took various steps to minimize the threat of COVID-19 to their congregations' health (20). On the other hand, mixed results were reported regarding the role that Chaplains played during the pandemic (21).

COVID-19 placed a significant burden on all levels of Israeli society. As of November 11, 2022, there have been over 4.6 million Israeli's infected with COVID-19 and 11,788 deaths (22). In general, mortality rates from COVID-19 in Israel were lower than those reported in the USA and some European countries (23). This is largely due to Israel's early response to the crisis, and a series of containment policies, at both national and local levels, including three national lockdowns, social distancing, mask wearing and closure of businesses and schools. The pandemic forced many events to be canceled. Notwithstanding the closure of wedding halls, weddings took place in private homes with the limitation of no more than 10 participants in each room; The Al-Aqsa Mosque and Dome of the Rock closed to prevent contamination of the holy sites (24).

Israel played a key role during the COVID-19 crisis as it was one of the countries that responded to the crisis early, and particularly among the first countries worldwide to vaccinate its' population. By the end of December 2020, Israel had begun a highly successful vaccination rollout. The lessons learned from Israel's experience and the challenges it faced helped other countries to assess their own situation and make adaptations accordingly. Israel is a multicultural country characterized by wide diversity. The Arab minority comprises 21% of the total population of whom belong to different faiths, Muslims (82%), Christians (9%), and Druze^1^ (9%) (25, 26). Recognizing that Arab religious leaders in Israel are well-respected and influential among their community, they had the potential to play a key role in promoting health during the COVID-19 pandemic. In Israel, to the best of our knowledge, this is the first study that has been conducted to examine the role of Arab religious leaders during the COVID-19 pandemic. In addition, previous studies only examined perspectives of religious leaders. This is the first of its kind to focus on decision makers as well as religious leadership. The aim of this study was three-fold: first and foremost we wanted to gain a deeper understanding of the role that key Muslim and Christian leaders in Israel played in responding to the pandemic; second, we aimed to assess the impact that their work had on the community; and finally we wanted to explore how this experience in Israel could serve as a model for how religious leaders can be engaged by health professionals and policy makers in times of crisis, which may be helpful in other contexts around the world, particularly in working with minority populations.

## Methods

### Study design and settings

We conducted qualitative research that included in-depth, semi-structured interviews with both Muslim and Christian religious leaders and Arab health policy makers from October 2021 to February 2022. Interviews were held in Arabic, either on zoom or in person, depending on the preferences of the individual and the COVID-19 situation at the time.

### Study population, sample size, and sampling

The research used purposeful sampling to recruit 17 participants (see Table 1) who played an important role in the community during the COVID-19 pandemic (27). The research population included 10 Muslim Sheikhs and three Christian clergy. The Muslim population in Israel comprises 82% of the general Arab population; hence, more Muslim religious leaders were recruited to represent the larger Muslim population. Four Arab health policy makers who played integral leadership roles during the COVID-19 crisis and worked directly with at the Ministry of Health were also recruited to participate in the study. We included these health policy makers to obtain their perspective regarding the role Arab religious leaders played and the impact they had on promoting health during COVID-19, and their beliefs regarding future partnerships. All participants were Arab men. The participants come from a range of areas across the country, representing different religious sub-groups within the Muslim faith.

**Table 1:** Demographic characteristics of sample population.

	**Age**	**Religion**	**Geographic region**
**Health policy makers**
Health policy maker 1	48	Muslim	Center
Health policy maker 2	62	Druze	North
Health policy maker 3	53	Muslim	North
Health policy maker 4	70	Christian	North
**Religious leaders**
Religious leader 1	68	Muslim	South
Religious leader 2	59	Muslim	South
Religious leader 3	74	Muslim	Jerusalem
Religious leader 4	68	Muslim	Jerusalem
Religious leader 5	47	Muslim	Center
Religious leader 6	62	Muslim	Center
Religious leader 7	49	Muslim	North
Religious leader 8	51	Muslim	North
Religious leader 9	47	Muslim	Mixed city
Religious leader 10	52	Muslim	Mixed city
Religious leader 11	53	Christian	National
Religious leader 12	48	Christian	North
Religious leader 13	62	Christian	North

### Research tools and data collection methods

In this study, two interview protocols were created for the target subpopulations: one for religious leaders and one for health policy makers. Interview protocols included a variety of semi-structured questions asking about the role they played during the pandemic, challenges they faced, and dialogue and partnerships they had. Table 2 details questions in the interview protocols. The protocols were pilot tested on one religious leader and one health policy maker for cultural appropriateness and sensitivity. Necessary adaptations were then made.

**Table 2:** Questions in semi-structured interview protocols for target populations.

**Topic**	**Target population**	**Questions**
Role/Position in community	Religious leaders and healthy policy makers	•Please describe your role/position in the community. •What organizations/institutions do you typically cooperate with? •Before the COVID-19 pandemic, how were you involved in promoting health among your community, if at all?
COVID-19 activities	Religious leaders and healthy policy makers	•Can you tell us about the specific activities that you have implemented in your community in response to the COVID-19 pandemic? What activities did you implement that were successful? What was not? How would you change/improve? •How did you view your role during the COVID-19 pandemic? How did your role change? How did you think the community perceived your role before and during the pandemic? •What challenges did you face during the COVID-19 pandemic?
Conflicts	Religious leaders	•Did you encounter various conflicts between religion and the COVID-19 guidelines set forth by the Ministry of Health. If so, what? •Were you able to find solutions/ways to cope and what specific issues could you not find solutions for? •Did you feel that the guidelines were closed and limited?
	Policy makers	•Did you encounter conflicts between promoting the health guidelines of the Ministry of Health and your work and religious leaders/general community? •Were you able to find solutions/ways to cope and what specific issues could you not find solutions for? •How do you see religious leader's role in promoting health promotion? •What difficulties/challenges/conflicts did you face when you worked with religious leaders (and general community)? •Was it difficult to approach religious leaders to discuss following the COVID-19 guidelines because they did not agree with different aspects?
Partnerships	Religious leaders	•Did you have any interaction, dialogue, or did you partner with religious leaders from other religions in terms of how to respond to the pandemic? If so, can you describe in more detail? •Did you learn something from religious leaders from other sectors/communities?
	Health policy makers	•Who were the key players/individuals that you were in contact with during the pandemic?
Dialogue	Religious leaders	•Can you describe any dialogue that took place between you and decision makers? •What difficulties/challenges/conflicts did you face when you worked with policy makers? •Did you feel comfortable approaching decision makers? •Did you have enough opportunities to engage with and reach decision makers during the pandemic?
	Health policy makers	•Can you describe any dialogue that took place between you and religious leaders? What difficulties/challenges/conflicts did you face when you worked with religious leaders (and general community)?
Work with the public	Religious leaders and health policy makers	•Was it easy for you to approach the public/general community to promote COVID-19 guidelines/vaccination/ etc? •Did you have enough opportunities (availability/accessibility) to reach the public and discuss the COVID-19 guidelines? •As the pandemic progressed, how do you perceive the community changed/responded to your work?
Trust	Religious leaders	•We know that often times trust among the Arab community toward governmental bodies/institutions is low. It was often the case that representatives of the governmental institutions were Arab health professionals themselves; they were the ones giving the public answers and guidance. Some have shared that they often felt stuck in the middle between government institution and the public. Can you elaborate what your perceptions/experience was?
Media channels and communication	Religious leaders and health policy makers	•What media/communication channels did you use throughout the pandemic to engage with the community/promote guidelines/etc? •Whose role is it to pass information to the public- doctors, health professionals, municipalities, religious leaders, etc? •Do you feel the public should have a role in decision making processes?
Cultural competence	Health policy makers	•Were the programs and guidelines of Ministry of Health culturally appropriate/suitable for the Arab population? •What more could have been done to make the guidelines more culturally appropriate for Arab communities?

### Recruitment and the research process

After receiving the approval of the ethics committee at Haifa University (approval number 404/21), purposeful sampling was used to recruit the key religious leaders by the research team, with the help of a representative from Mosaica, a non-profit organization that has vast experience working in conflict resolution in the community, particularly religious leaders.

The inclusion criteria included Arab religious leaders from Islam and Christian faiths living in Israel. Views and perceptions can widely differ even among the same religious group. For example, the Islamic brotherhood in the North of the country is very different than in the South. Thus, we purposefully recruited religious leaders from the different groups and geographic regions to get a better perception of all views. A total of 15 religious leaders were approached by the representative from Mosaica who explained the research objectives. Two refused participation due to lack of time. The remaining 13 religious leaders consented to participation.

Two researchers from the team contacted *via* phone the Arab health professionals who worked directly with the Arab community during the COVID pandemic. They explained the objectives of the research, and set up interviews if they agreed to participate. All four agreed to participate. Participation was completely voluntary. All participants provided informed consent before participation in the interviews.

All interviews were conducted on zoom by the research team in Arabic and lasted between 45 and 90 min. The questions were open ended and probes were used to elicit more information. All data collected during the research was entered and stored on the researchers' computers in password-protected files. The information was treated confidentially and all those who handle the research materials were bound by a signed professional confidentiality. The results were reported in such a way that individual respondents cannot be identified.

### Data analysis

A research team of three researchers analyzed the interviews using thematic analysis framework following the guidance provided by Braun and Clark (28). The six-phase framework to identify key patterns in the data was used and included: (1) familiarizing with the data; (2) generating initial codes; (3) searching for themes; (4) reviewing of the themes; (5) defining and naming of themes; and (6) reporting the findings (28).

Relevant themes and subthemes were extracted concerning the role of the participants during the pandemic, challenges, partnership, communication platforms, and lessons learned. All data collected (interviews, recordings, lists, and notes) were transcribed, and translated into English, in order to be analyzed by the research team. The completed transcriptions were compared with hand-written notes. We coded all the data and anonymized removing any identifying information. To ensure reliability, two members of the research team carried out independent analyses of the data. Each began the analysis by reading the transcripts to extract general and potential meanings. Then, each created an initial coding structure based on descriptive coding resulting from coding units of text as themes by labeling them with a phrase related to the participant's account. In the next stage, we conducted a joint analysis and consolidated the identified themes (28, 29).

### Ethics

This study was approved by the Ethics Committee of the Faculty of Social Welfare and Health Sciences at the University of Haifa (approval number 404/21). All study participants gave their consent to participate in the research and publish its results.

## Results

A total of 17 in depth interviews were conducted with Muslim religious leaders (*n* = 10), Christian religious leaders (*n* = 3), and Arab health policy makers (*n* = 4) between October 2021 and February 2022.

**Figure 1:**
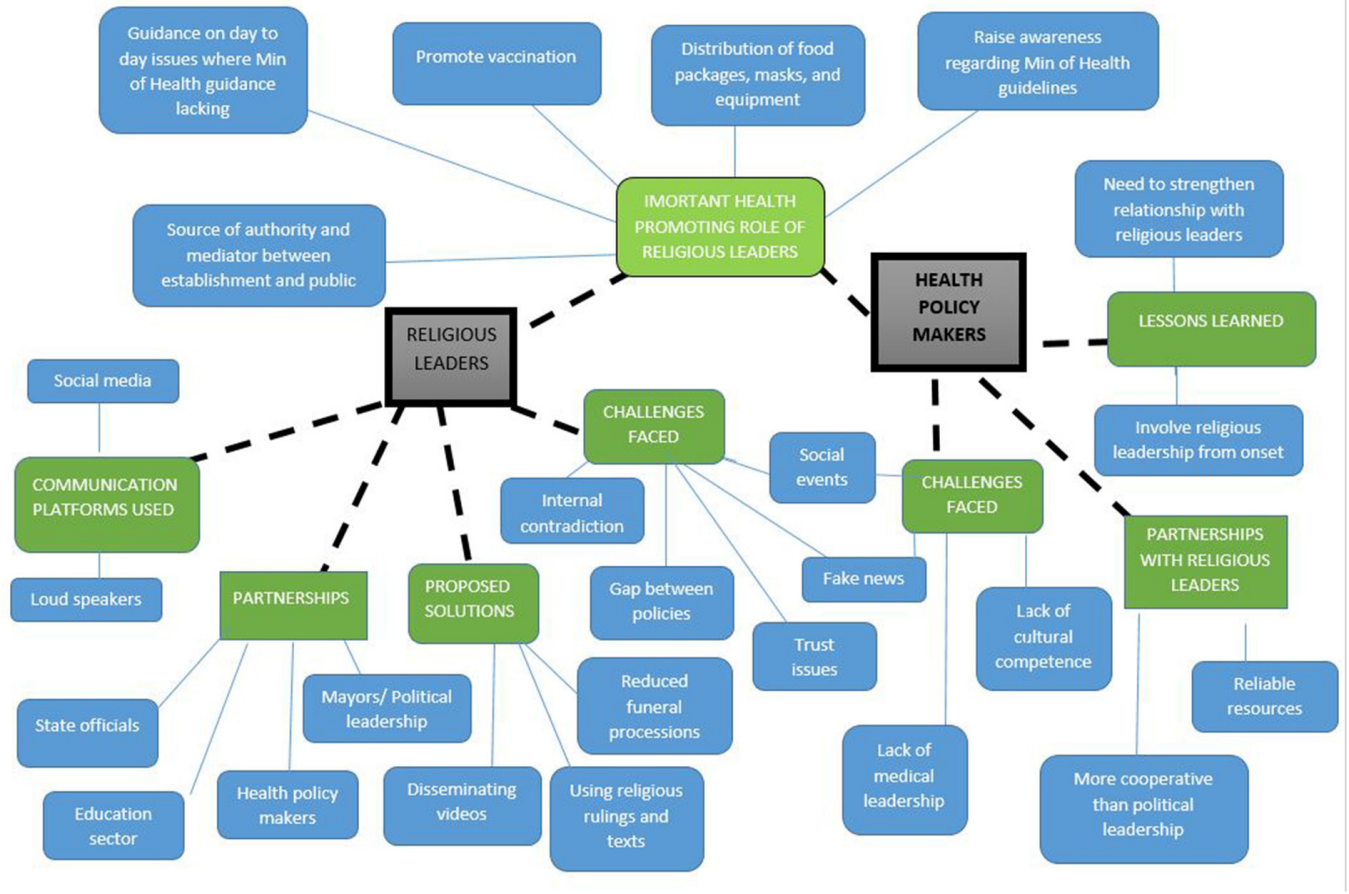
Thematic map of predominant themes.

There were a number of predominant themes that arose from the analysis (Figure 1).

### Predominant themes from religious leaders

#### Important role of religious leaders during COVID-19 crisis

The Arab religious leadership, both Muslims and Christians, played an integral role during the pandemic. All religious leaders interviewed implemented different health promoting activities during the pandemic. This included distributing food packages, masks, and protective equipment to those in need or families in quarantine, as illustrated by the following quote:

“Religious leaders worked to promote food security. We distributed food packages to the community and worked directly with municipalities. Usually, food packages were distributed in the Mosques to those who were in need and those in quarantine.” (Religious leader 10)

Religious leaders promoted vaccination and its importance and dedicated great efforts to reducing vaccine hesitancy among their followers. They were role models, often being the first to get themselves and their families vaccinated, and many shared videos and pictures of receiving the vaccine to encourage their followers to do the same. This is illustrated by this quote from one of the leaders:

“At first there was a lot of hesitancy to get vaccinated- and fear…. I could understand people's fears. It was new and everything happened so fast. When people came to me and asked what my thoughts were…. I promoted the vaccine and found religious writings that support vaccination. When I went to get all the vaccines, I took my family and took a picture getting vaccinated and shared them. This made people see that I wasn't just talking- I was actually following the recommendations myself.” (Religious leader 12)

Religious leaders worked to raise awareness regarding the Ministry of Health guidelines, including social distancing, proper hygiene, and limiting social gatherings. This was also enforced by religious leaders in the mosques and churches. Many religious leaders called for people to pray at home to prevent gatherings and even closed the mosques and churches. During periods of lockdowns, prayer services went online.

“The Ministry of Health cooperated with the religious leaders in the Mosques to disseminate the guidelines because they were the trusted body in the community”. (Religious leader 4)

As a source of trust among the public, religious leaders often served as an authority and the general public approached them for guidance and leadership. Finally, they were also involved as mediators between the establishment or the state and the public. They played a significant role in providing guidance to the Ministry of Health on day to day issues that they dealt with but where the ministry of health was absent in providing guidance. One example of this had to do with bathing the deceased, which is illustrated by this quote:

“No one was thinking about the procedures related to washing the deceased bodies. We didn't know how to act or deal with those individuals who died from COVID-19- are we allowed to approach the body, be near them, are they still infectious. There was no communication or guidelines regarding how to deal with this and we didn't know what to do. In the beginning, we didn't feel like there was respect for the dead; but after many approaches from us to the Ministry, they finally decided to open up centers especially dedicated to washing the deceased bodies of those who passed from COVID-19.” (Religious leader 5)

#### Challenges faced by religious leaders during COVID-19

There were a number of challenges that the religious leaders reported in implementing their work.

There was often a gap between policies for the religious institutions and other places, which was a source of major frustration. This is illustrated by a quote from one of the Muslim religious leaders:

“At the time that we started closing the Mosques, everything else was still open- parks, beaches, malls, etc. It was hard for us to explain to the public why we had to close our doors when everything else was opened. In the end, we were able to convince the public why we were closed because we described to the people that our reference is profit Mohammad and not the Ministry of Health. This caused people to listen to us. (Religious leader 6)

Both Muslim and Christian leaders emphasized Changes in risk perceptions and compliance as waves progressed. In the beginning, there was a sense of fear due to the lack of knowing want to expect and the public listened to them more and followed the guidelines more strictly. As the pandemic progressed, many started to take the virus less seriously and they felt they had less of an impact than in the beginning waves. One Christian leader stated:

“There was always trust and respect toward me, but as the pandemic progressed, and COVID-19 was not new anymore, I felt people respected what I said and promoted, but it wasn't like the beginning.” (Religious leader 11)

Internal contradictions that the religious leaders felt was another challenge reported. In the past, they were always urging people to gather together to pray- the faithful should congregate and come to places of worship. Now, suddenly they had to tell their followers not to come to the “house of God”. It was very difficult and unnatural for them.

“We also had religious dilemmas due to the contradiction between the belief of Islam to gather in large crowds to pray together and what we were telling people now to pray in their homes. In addition, I couldn't allow in 400 meters space only 20 people to enter if 200 arrived to pray. I couldn't be selective in deciding who to enter- the elderly or the young. (Religious leader 1)

Probably the biggest challenge in Arab society was limiting social events. In Arab culture, weddings, engagements, funerals are all large, important, social events. Families come together for days to celebrate. As religious leaders, they had responsibility to perform the religious ceremony. However, they encouraged people to not have parties and limit events, but often times the public didn't listen.

“As the pandemic progressed, very few listened to our recommendations to not hold big weddings and events- only about 2%. Some people tried to reduce numbers- but parties/events were still happening. In our Arab society- you know- the weddings and parties start from a week or more before. Some people cut down to only the church ceremony and 1 big party- but even this had a lot of people. Some groups had the church ceremony and a small reception but after the first wave people went back to their old ways, despite our recommendations.” (Religious leader 11)

Dealing with fake news was another real challenge. It was the case that religious leaders or more accurately, religious scientists, often had to issue fatwas or religious rulings to convince people that news that came out was fake- particularly when dealing with the conspiracy theory.

“There were so many things that were not true that were published on social media and people believed it. Because COVID-19 is something new- at first it was hard for people to understand what to believe and what not to. I even had people who I knew and that trusted me tell me- they even have you believing that there is such a thing as COVID-19- its all made up by the politicians of the world and by the pharmaceutical companies who just want to make money”. (Religious leader 5)

Trust issues (or rather lack of trust) among the Arab community was another challenge. There was a lack of trust in the State by the general public. Many religious leaders indicated they sometimes felt “stuck” in the middle between the government institutions and the public. They reported they sometimes felt discrimination in the application of the policies, as illustrated by this quote:

“ I felt this most when it came to the police coming to enforce the regulations. I felt stuck in between knowing that the policy and guidelines needed to be enforced and between the basic resident who was in a sense also not really aware. When the police came, it caused a lack of trust and sometimes even violence.” (Religious leader 8)

#### Solutions of religious leaders to deal with challenges

Religious leaders came up with a variety of solutions to deal with the challenges and issues they faced. One area they were successful was to reduce funeral proceedings to limit gatherings. The actual funeral ceremony was limited to immediate family and they were able to move paying condolences to WhatsApp and online (Facebook). A Muslim religious leader shared:

“In each Mosque, there is an area for mourners. We as religious leaders closed these to promote people from gathering. We asked people to try to support and offer their condolences over the phone, rather than visiting in person. During the funerals, we prayed over the deceased in the cemetery, not in the mosque. This was very hard for the family.” (Religious leader 3)

To deal with fake news, religious leaders made videos and disseminated them through social media to dismiss myths. One religious leader stated:

“To deal with fake news that was being quickly passed around through Whatsapp and other social media sites, I also had to act quickly. This often meant reaching the younger generation who had more access to these platforms. I would often make videos myself urging people not to listen to what was being said about the vaccine and spread the true facts.” (Religious leader 5)

Senior religious leaders wrote rulings and used religious texts to convince people regarding the importance of following the ministry of health guidelines. One quote that illustrates this:

“Vaccination was the biggest challenge in the pandemic because of the conspiracy theory. Some people believed that the State wanted to destroy the Arabs through vaccination. Others believed the pandemic was a global conspiracy to decrease the number of people on Earth. These two beliefs forced religious leaders to bring from the Koran the religious writings that said human life is the most important thing.” (Religious leader 6)

#### Communication platforms used by religious leaders

Both Muslim and Christian religious leaders noted that the COVID-19 pandemic forced them to use different communication platforms to reach their followers. Many used social media and digital platforms, including Facebook, Instagram, and Whatsapp (and some used Tiktok to reach the younger audience). These were innovative digital platforms that religious leaders did not use before. They recognized the potential these platforms had, but also the dangers in use. One religious leader shared:

“I used social media, particularly facebook, a lot in my work to promote the guidelines. I posted videos to encourage mask wearing, praying at home, avoiding large gatherings, but it was also my direct source of connection with my followers. I recorded the prayers and the public could join live and pray with me directly. It was a tool that was used to promote the positive. At the same time, unfortunately, it was also a tool that was being used to spread fake news. It gave an easy platform for anyone to spread anytime of message that they want. Within seconds, you could get something out that wasn't true. So we need to be cautious… it can be used for good- but it could also be a dangerous bomb waiting to explode. (Religious leader 11)

Especially in the South of the country, religious leaders started to use the loudspeakers in different neighborhoods as a way to raise awareness regarding the Ministry of health guidelines and encourage the public to physically distance, avoid gatherings, wear masks, and get vaccinated.

“We took advantage of the loudspeakers, we reached all neighborhoods. We called on people to follow the guidelines. We made announcements to the people, we reached everyone.” (Religious leader 3)

#### Partnerships with various professionals

Both Muslim and Christian leaders emphasized the importance of partnership with other bodies as part of their work. This involved working together with mayors and municipality members, health professionals, and the educational sector. There was also cooperation between different religious leaders- of the same religion- as well as between religions, in which they shared experiences and learnt from one another. This theme is illustrated from the following quote:

“We were in contact with the heads of local authorities, sheikhs, and doctors *via* Zoom. We published fatwas to encourage vaccination, and we were in full contact with the Arab Society Office in the Ministry of Health to raise awareness and give answers to inquiries.” (Religious leader 7)

Interestingly, some religious leaders had behind the scenes conversations with security and State officials to provide insights to the government on how they should work or approach the general public in a way that is culturally sensitive to the needs of the population. However, the majority of religious leaders felt they did not have enough opportunities to engage with decision makers who were higher up.

### Predominant themes from health professionals

#### Role of religious leaders during COVID-19

All four health professionals that we interviewed emphasized that both Christian and Muslim religious leaders played a significant role in helping combat the spread of the COVID-19 virus and protecting the health of the public. These roles included raising awareness regarding the Ministry of Health COVID-19 guidelines, reducing morbidity by closing religious institutions, promoting vaccinations by serving as role models, and providing advice and guidance on issues that professionals could not. For example, one health policy maker stated:

“What was not easy were the religious issues that we as professionals could not solve such as the issue of washing the dead or canceling Friday prayers or Ramadan prayers, or Sunday prayers in churches and more, we were only giving advice. It was the role of the religious leaders to decide how things needed to be properly done.” (Health policy maker 1)

#### Partnership with religious leaders

All health professionals indicated that the religious leaders were a key partner. They served as a reliable resource and source of information. Because they were very powerful and trusted among their followers, health professionals felt that religious leaders were good mediators between the Ministry of Health and the public. In general, the health professionals even felt that the religious leaders were more cooperative than the political local Arab leadership- who often times didn't enforce regulations or guidelines or give fines as they should for fear of losing political support. A quote that illustrates this is:

“I can only tell you that from my experience the religious leaders were very positive and respected the guidelines and recommendations of physicians… much more than politicians. I of course didn't meet with all religious leaders, but the ones that I did meet with were very supportive, cooperative, and following recommendations- and urging others to do so”. (Health policy maker 4)

#### Challenges health professionals faced during COVID-19 crisis

Health policy makers also faced many challenges, some similar to those of the religious leaders. The challenges that were similar included: Dealing with fake news and the conspiracy theory, social events and gatherings, dissonance between outbreak and easing of policies as waves progressed, and distrust of government by Arab community. Other challenges reported by health professionals included the non-response of specific groups to following the guidelines- particularly the younger population who felt that even if they were infected with COVID-19 it would not impact them.

Another challenge they indicated was the lack of medical leadership in the Arab community to serve as role models for the general public. Of course some Arab physicians were leaders, but many were not. This is illustrated by the quote:

“In Arab society- doctors are seen as role models and people do what they say and copy what they do. We have a responsibility. But when you see doctors attending wedding parties without a mask, it sends a bad message to the public. They should be leaders and role models…”. (Health policy maker 4)

Some of the health professionals indicated that there was a lack of cultural sensitivity and understanding of the Ministry of health guidelines, as indicated by this health policy maker:

“I think most aspects set in the Ministry of Health guidelines were not culturally appropriate- particularly in the beginning. There is no awareness of cultural competence. Too many in the ministry and higher up, to them cultural competence is simply translation to Arabic- and this is not the case. The decision makers making decisions don't understand this and still don't… even the people higher up”. (Health policy maker 4)

A major issue was the lack of enforcement by Arab political leadership of guidelines and fear of local enforcement. Many Arab mayors were not willing to issue fines because they feared either losing political support or more seriously, feared violent actions would be taken against themselves or the families.

“The difficulty and challenge was enforcement of policies in the Arab towns. This could not be done by the local mayors. They even told me- you want me to stop a wedding party and fine the family- how do I know with such violence increase in our society they will not come and send someone to shoot at my car, my home, or my family. In my opinion, this was not dealt with properly by the government. I don't really have a solution- because if you send in police it will also cause increased tension and anger toward the state”. (Health policy maker 2)

#### Lessons and areas for improvement

Health professionals raised several lessons learned and areas of improvement in working with religious leaders. First, they agreed that there is a need to strengthen the link between religious and professional leadership and consult and involve them more frequently, both during times of crisis and on a regular basis. They also emphasized that religious leaders need to be involved from the beginning in decision making processes. This is evident from the following quote from one of the health professionals:

“I think we could have been in stronger contact with the religious leaders, and benefit more from their impact on the public. I think we didn't do it enough. It was more on occasions and holidays, when the situation was serious, so when we wanted to convey the messages, we did. It is through them, which is unfortunate. For example, they were not with us on any of the committees, the idea is that they should be more involved and partners in every step.” (Health policy maker 2)

## Discussion

According to the literature, religious leaders can have a great influence and impact on promoting health, particularly during times of crisis. There is limited empirical research that has examined the role religious leaders had during the COVID-19 pandemic, particularly in Israel. In our study, we examined the in-depth role that Arab Muslim and Christian religious leaders in Israel played to promote health during the COVID-19 crisis. Religious leaders had a powerful impact on the community and served as an important bridge between the community, health professionals, and State institutions. Furthermore, this important role and impact was recognized by Arab health professionals.

Similar findings related to the powerful impact of religious leaders in combatting the COVID-19 health crisis has been reported (30, 31). Another study that examined reducing mask resistance among White evangelical Christians found that messages from religious leaders on mask use were more effective than messages from the government or patriotic messages (32). In Pakistan, religious leaders repeatedly urged people to follow the safety norms provided by the health department, especially to stay at home (33). Research conducted among the Muslim community in the United Kingdom found that religious texts were important in the context of promoting COVID-19 guidelines, such as closing Mosques (34).

Several studies in the literature have examined faith leaders' role in promoting the COVID-19 vaccination and working to reduce vaccine hesitancy. One study in Nigeria emphasized that religious leaders significantly influenced COVID-19 vaccination rates (35). A study conducted by Viskupic and Wiltse (36) that surveyed 709 residents from South Dakota, United States found that religious leaders had a positive and statistically significant effect on them getting vaccinated against COVID-19, in comparison to messages from medical or political leaders that had no statistically significant effect (36). Their results strongly suggest that religious leaders are more effective messengers than other potential messengers and that public health officials would be well served to coordinate their efforts with leaders in faith communities. Particularly in relation to COVID-19 vaccination, the importance of collaboration with religious leaders and multi-sectoral partnerships is important in reducing hesitancy and work toward the shared goal of building public trust in vaccines (37). A systematic review published in May 2022 found mixed evidence that religious leaders can both promote vaccination and inhibit vaccination, suggesting the need to engage religious leaders more effectively (38). Another study in 22 countries across Europe showed that in countries where the majority religious group had a leader who publicly and consistently supported vaccination against COVID-19, religiosity was positively related to vaccination rates. Conversely, in countries where the leaders of major religious communities had a neutral or an unclear position toward vaccination, religiosity remains negatively related to the vaccination rate at the country level (39).

On the other side, religious leaders in some countries played a negative role. COVID-19 was viewed as God's wrath by some religious leaders in Ethiopia, and in Pakistan, some people considered COVID-19 as a part of the government's religious propaganda to keep them under pressure (40). In Iran, some religious leaders did not accept the closing of religious institutions, believing that the religious institution is a “house of healing” and closure would cause more harm that the consequences of COVID-19 (41). This internal conflict within religious leaders between telling followers to come to the house of worship and having to close the religious institutions was a major challenge reported by religious leaders in our study. Other challenges they faced included dealing with fake news, dealing with social events and gatherings, gaps in policy toward religious institutions, and lack of trust toward State.

We found complex findings regarding religious leaders' modes of influence and their ability to influence. Both Muslim and Christian religious leaders in Israel were successfully engaged in health promoting activities during the pandemic, including distribution of food packages, masks, and protective equipment to those in need or quarantine, vaccination promotion, and limiting social and religious gatherings. However, the impact of their work differed depending on the specific target population they worked with. For example, both Muslim and Christian religious leaders reported their activities had less of an impact on the younger generations in comparison to work with the older (elderly) populations. The elderly tend to be more conservative in their behavior, have increased fear of death, and more difficulty accessing health services. The young, who are mostly a healthy population group, were less likely to be concerned about catching the virus and expressed less concerns. In addition, they are more exposed to global controversy through social media, and have different role models. To reach this audience, religious leaders turned to innovative media platforms and new modes of communication, such as Instagram and Tiktok, which was something they had never used before. It is also important to note that the power of impact of the religious leaders was much greater in the beginning of the pandemic, when there was still much unknown about the virus and increased concerns among the general population.

The influence of the religious leaders was also a derivative of the level of involvement that the health policy makers shared with them. The majority of religious leaders were not involved in the actual policy making process from the beginning, rather only became involved in promoting the instructions and guidelines after they were established. Religious leaders became involved when policy makers recognized that they needed to cooperate with them as trusted gatekeepers in the community. As a result, the essential feedback and input from a religious perspective was not taken into account from the onset in the development of guidelines, which was a source of conflict and often created tensions, frustrations, distrust, and antagonism. This created a sense among religious leaders of feeling “stuck in the middle” between the government institutions and the public. This was also recognized by health policy makers, who emphasized the key role religious leaders had in promoting health during COVID-19, and stressed the importance of engaging religious leaders from the beginning in decision making processes; not only to give them instructions to pass on to their followers- but to include them.

Another important finding of the present study raised the question of who is the religious leader and what is their role? COVID-19 led to an update in the role of the religious leader; before COVID-19 religious leaders dealt mainly with religious issues whereas during the pandemic a new vision was proposed regarding their role and they created multi-sectorial partnerships for improving their community's health. The importance of such multi-sectorial partnerships for improving the community's health and emphasizing the importance of the collaboration of science and religion has been emphasized in the literature (33, 42).

From the interviews, it was also evident that there was a difference between religious leaders who are involved in the political sphere and those who are experts in religious laws. Not all religious leaders can issue Fatwas or religious rulings. To avoid conflicts, it is necessary to understand the different roles and the experts in religious law be consulted to provide their input and perspectives.

### Study limitations

There are some research limitations that need to be considered. First, since this is a qualitative study using purposeful sampling, it is therefore not a representative sample. Nevertheless, we religious leaders from different geographic locations throughout the country and from different religious sub-sects to get a better understanding of possible differences in perceptions and activities. Another limitation is the small sample size. We only interviewed 13 religious leaders. This is not an easy target group to reach and interview. It is important to note that this group has great value in terms of the information it raises. Despite the small sample it must be acknowledged that thematic saturation was achieved. Another limitation was the lack of female representation in our study sample. Although female Muslim religious leaders are non-existent and Christian female religious leaders in the area are very few if at all, there are female health professionals, policy makers, and doctors working in the field. Future research should include female health professionals in their study sample. Follow-up studies looking at religious leaders from different communities in the region, including Druze and Jewish populations, or comparative studies with populations from other countries are needed. Furthermore, a follow-up study on the role of the religious leaders in routine life after the COVID-19 pandemic.

### Conclusions and future recommendations

Muslim and Christian religious leaders in Israel played a pivotal role during the COVID-19 pandemic. These religious leaders have shown unprecedented unity by working together for the health of all, regardless of faith or ethnicity. They served as a powerful bridge between health authorities and communities and were effective as mediators who reconcile science, policy and religious perspectives. They can also serve as health promoters within their communities who can identify the unique needs of the people, reach vulnerable groups, and act as respected, credible influencers. As public health professionals it is important to engage religious leaders not only in health promoting activities during times of crisis, but also during policy making.

Based on the findings of this study, there are a number of policy recommendations for moving forward. Religious leaders have the potential to be key players in promoting health during times of crisis. To strengthen their impact, it is important that they be integral and integrated players from the very beginning, in the early times of crisis, in all aspects related to the design and development of health guidelines. Their contribution can enhance the cultural and religious acceptance and appropriateness of health guidelines, which will increase the likelihood of implementation by the general public. When working during health crises, it is critical to separate religion and politics to minimize conflicts and conflicts of interest. Health policy makers and professions should consult with religious leaders who are experts in religious laws, and not only those religious leaders involved in the political world. In addition to religious leaders, health professionals should lead multi-sectorial interventions to involve as many partners as possible to reach wider impact.

## Data availability statement

The raw data supporting the conclusions of this article will be made available by the authors, without undue reservation.

## Ethics statement

The studies involving human participants were reviewed and approved by the Ethics Committee of the Faculty of Social Welfare and Health Sciences at the University of Haifa (approval number 404/21). The patients/participants provided their written informed consent to participate in this study.

## Author contributions

JE-H and DR co-led conceptualization of the study. JE-H, NAES, DR, and AG-E made substantial contributions to the design of this study. JE-H and NAES collected, analyzed, and interpreted the data. JE-H led manuscript preparation. JE-H, NAES, and AG-E contributed to data interpretation and writing of the manuscript. All authors contributed to the article and approved the submitted version.

## Footnotes

^1^The Druze are a religious and ethnic minority among Arab citizens of Israel. In 2019, there were 143,000 Druze living in Israel and the Israeli-occupied Golan Heights, 1.6% of the total population of Israel and the Golan Heights. Even though the faith originally developed out of Ismaili Islam, Druze do not identify as Muslims. In 1957, the Israeli government designated the Druze a distinct ethnic community at the request of its communal leaders. The Druze are Arabic-speaking citizens of Israel who serve in the Israel Defense Forces. They live mainly in the north of the country. Israel has the world's third largest Druze population, just after Syria and Lebanon.
